# Cloud based evaluation of databases for stock market data

**DOI:** 10.1186/s13677-022-00323-4

**Published:** 2022-09-29

**Authors:** Baldeep Singh, Randall Martyr, Thomas Medland, Jamie Astin, Gordon Hunter, Jean-Christophe Nebel

**Affiliations:** 1 School of Computer Science and Mathematics, Kingston University, London, KT1 2EE UK; 2 Instinet Global Services Limited, 1 Angel Lane, London, EC4R 3AB UK

**Keywords:** Big data, Distributed storage, Cloud databases, Hybrid cloud, OHLC data

## Abstract

About fifty years ago, the world’s first fully automated system for trading securities was introduced by Instinet in the US. Since then the world of trading has been revolutionised by the introduction of electronic markets and automatic order execution. Nowadays, financial institutions exploit the associated flow of daily data using more and more advanced analytics to gain valuable insight on the markets and inform their investment decisions. In particular, time series of Open High Low Close prices and Volume data are of special interest as they allow identifying trading patterns useful for forecasting both stock prices and volumes. Traditionally, relational databases have been used to store this data; however, the ever-growing volume of this data, the adoption of the hybrid cloud model, and the availability of novel non-relational databases which claim to be more scalable and fault tolerant raise the question whether relational databases are still the most appropriate. In this study, we define a set of criteria to evaluate performance of a variety of databases on a hybrid cloud environment. There, we conduct experiments using standard and custom workloads. Results show that migration to a MongoDB database would be most beneficial in terms of cost, storage space, and throughput. In addition, organisations wishing to take advantage of autoscaling and the maintenance power of the cloud should opt for a cloud native solution.

## Introduction

The first stock exchange was established in the 1600s by the East India Company [72]. A stock exchange was a building where existing and prospective investors met to buy or sell shares. Open outcry was the main method of communication on a trading floor. It involved shouting and hand gestures to transfer information about the orders. That model remained more or less unchanged for centuries. With the introduction of internet connectivity and more powerful computers in the late 1980s and early 1990s, the push towards automation overtook the holdovers from open outcry. In the early 2000s a seismic change in trading mechanics began, with the increased use of electronic trading. By late 2019 about 80% of the cash equity markets were all electronic [82].

Advances in computer technology has led to faster order execution, less human error, and greater ability to carry out research on the market. Trading, nowadays, relies on constant and incredibly fast analysis of very large amounts of data [77] which is often in time series consisting of a date, a unique identifier (such as a stock ticker), and values observed that day for an entity. One such type is the OHLC data which records the Open, High, Low, Close prices^1^ of an instrument in a given period of time.

OHLC data are particularly important for the derivation of patterns and trading signals from market data [64, 66]. Indeed, Fiess and MacDonald [30] consider that the conventional wisdom behind recording these prices rather than all intra-daily prices is their higher informational content. OHLC data can be used to define and forecast asset price volatility [30, 63] and are often less costly to obtain and work with than the high frequency tick data which consists of bid and ask prices aggregated from multiple exchanges [63]. In fact, [63] shows that volatility models built on daily OHLC time series data may provide similar accuracy to those built on high frequency data. Consequently, investors continue to make purchases and sells according to accurate predictions of OHLC data [93].

A system for storage and retrieval for time series data—a time series management system or a time series database—is necessary to conduct such analyses [46].

As OHLC data are typically generated in the application layer by a program (or a script) that processes measurements of the upwards or downwards price movements of stock [7], it is very important to store this data in a time series database where it is easy and fast to store, query, and perform operations, such as sum, mean and median, on multiple records of data. Moreover, since financial time series databases can very quickly grow very large — there are several thousand stocks listed on the New York Stock Exchange alone, it is critical to have an efficient database solution able to perform the required large-scale analytics processing. As traditional database solutions such as Relational Database Management Systems are typically sub-optimal and generally ill-suited for time series data [78], several new solutions have been proposed [46].

Even though these time series databases seem promising, they do not have a wider support like SQL (Structured Query Language) or NoSQL (Not Only SQL) databases and are typically very hard to migrate to.

In this paper, we investigate and design a widely prevalent, scalable and distributed database system for storing financial stock market OHLC data achieving high throughput in a cost-effective way. Such a system should allow financial institutions to store large volumes of incoming data on a distributed network while still being able to obtain the query results with minimal latency. In our experiments we use historical OHLC data to compare the performance of different types of databases. The main contributions of this study can be summarised as follow:

1. An extensive literature review that includes both theory- and experiment-based comparisons of relational and non-relational databases.
2. A set of criteria to perform holistic evaluation of the ability of a database system at storing and querying OHLC financial data on a hybrid cloud deployment architecture.
3. A comprehensive set of experiments using the “Huge Stock Market Dataset” to assess performance of the most popular relational and non-relational databases according to the criteria previously defined.

The remainder of this article is organised as follows. First, the ‘State-of-the-Art Review’ Section discusses the development of database systems, their features, and their performance in published case studies. Second, in the ‘Hybrid Cloud Architecture for Secure and Efficient Storage and Processing of Financial Data’ Section where the focus is on applications relying on OHLC price data, not only is a set of important criteria introduced to help the selection of the most suitable database, but also an architecture is proposed to conduct the experiments required to test these criteria. Third, the ‘Experiments and Results’ Section starts with the presentation of the environment, data, evaluation framework and scope; then it reports the results of the experiments, which eventually leads us to identify which type of database satisfies our application of interest. Finally, the last Section concludes the paper and suggests future research directions.

## State-of-the-art review

This section first reviews the development of both relational and non-relational databases and highlights their respective strengths. Second, it analyses the outcomes of experiments conducted on various case studies to assess their individual performance. Finally, as the main area of interest of this study is the usage of databases to store and process large volumes of financial trading data, usually on a hybrid cloud architecture, this review explores then their storage costs and the characteristics of their cloud implementation.

### Definitions and theory-based comparisons

In 1970, Edgar F. Codd proposed a new model of data called relational database where all data are represented in terms of tuples and attributes, formally described using tables [21]. The platforms used to manage these databases are known as Relational Database Management Systems (RDBMS). Most of them employ SQL (Structured Query Language) as their query language [13]. Relational databases rely on the ACID (Atomic, Consistent, Isolated, Durable) properties to operate efficiently and correctly [34]. This guarantees data validity despite errors, power failures and other mishaps [37].

Relational databases perform best with structured data, but they have a limited or restricted ability to represent complex semi-structured or unstructured data [55]. A study has shown that it is difficult to store clinical visit data in an RDBMS due to their semi-structured information and dynamic changing properties [94]. Indeed, usage of relational databases for such data leads to creating fields that are mostly empty resulting in inefficient storage and poor performance. Moreover, another limitation of relational databases is their inability to store increasing volumes of real-time data [10]. As in the cases of national votes and fingerprints data, the amount collected increases drastically both in terms of volumes (Terabytes of data) and velocity (rate of data generated, in Gigabytes/day), which eventually requires a large number of tables to accommodate the growth in data. Actually, the usage of a relational database in such scenarios becomes inappropriate because of its inability to scale with the ever-growing real-time data [83].

Finally, relational databases cannot take advantage of modern advancements in distributed computing as they are not designed to function with data partitioning [95].

The non-relational databases were created as a means to offer high performance (both in terms of speed and size) and high availability at a price of losing the ACID trait of relational databases and instead offering the weaker BASE (Basic Availability, Soft state, Eventual consistency) feature [12, 52]. These databases store semi-structured and structured data in a non-complex data model such as key-value pairs, which consists of two parts, a string which represents the key and the actual data which is referred to as value. These keys are then used as indices, making the query process faster than the RDBMS [50]. Non-relational databases started becoming popular with the internet boom in the mid-1990s as relational databases could not handle the flow of information demanded by users [41]. Since then, numerous companies and organisations have developed their own non-relational databases [67, 87].^2^ Many studies have shown that non-relational databases enable better performance in terms of speed and flexibility [36, 38, 89]. Indeed, availability, real-time response, advanced data analysis, and the ability to manage bigdata remain weaknesses which are displayed by relational databases [28]. Moreover, these shortcomings are overcome by the latest NoSQL systems which have been designed to address the challenges associated with dealing with large amounts of data [76]. As a consequence, they have become the option of choice for applications involving geographically distributed data, large amounts of data, or scalability requirements [47, 56, 88]. This is particularly the case for services relying on Internet of Things (IoT) technology [89]. For example, in a recent case study where IoT enabled sensors provide measurements to monitor manufacturing defects in the automobile industry, usage of a NoSQL database allowed real-time data processing and, thus, the detection of faults at early stages of the manufacturing process [33].

Unlike relational databases that can only scale vertically by adding more resources to the current server, non-relational databases also support and embrace horizontal scaling. This is achieved by adding more machines to the network and then dividing the workload or in this case distributing the data among them [83].

Despite this, the latest Database Engine rankings [23] (based on top searches on various search engines, Stack Overflow, Google trends, job offers or number of mentions in social networks) reveals that relational databases remain prevalent: there are only three non-relational databases in the top ten and none of them are in the top four! This is probably because relational systems have been used extensively for many decades and are trusted for maintaining accurate transactional records, legacy data sources [71, 99], and many other use cases within organisations of all sizes [48]. In addition, non-relational databases lack a standard query language [57, 60]: there are more than 200 implementations, each providing its own language and interface [20] that developers and users must learn. Finally, a major challenge of non-relational databases is their weak security mechanisms [6]. Indeed, they were initially designed without security being considered as an essential feature [81]. Thus, there have been growing concerns related to data privacy in NoSQL systems which results from compromises made for better performance and scalability [32]. Whereas relational databases have inbuilt authentication instead of relying of a middleware application for authentication or authorization of the data source, by design, non-relational databases offer limited security and place more emphasis on data handling [51]. Indeed, the feature of distributed data, termed as ‘sharding’ [9], which is considered the key of their success, is associated with a concern on how the confidentiality and privacy of data is maintained across systems [73].

### Experiment-based comparisons

Many experiments have been conducted to compare characteristics of non-relational and relational databases including their scalability, performance, flexibility, power of querying, and security [3, 17, 58, 68, 69, 70, 86]. Experiments conducted a decade ago proved quite inconclusive as performance varied significantly according to the type of operation performed and the type of data used [58, 86]. Focusing on processing a modest amount of structured data, it was shown that MongoDB – a popular non-relational database – performed at least as well as MySQL with exceptions of aggregate functions (such as medians, modes and sums) [69]. A more recent study analysing performance of non-relational databases for spatial and aggregate functions suggests that the performance of MongoDB has since improved [3]. Focusing on applications handling large volumes of data (i.e., terabytes), it was concluded that non-relational databases were preferable because they offer flexible architectures which can accommodate a large variety of data storage needs [68, 70]. Similar results were obtained in a performance comparison of various types of non-relational databases against MySQL [35]. Focused on the storage of unstructured data of hospital patients during COVID-19, various forms (Key-value stores, Graph based, Column-oriented, Document) of non-relational databases were evaluated based on their data model, CAP (Consistency, Availability, and Partitioning) theorem [31], suitability for being distributed across multiple servers and other factors [27]. The authors eventually designed an algorithm able to suggest the most suitable database type according to the hospital’s needs. Also targeting a COVID-19 dataset, a recent study investigated data retrieval from an unstructured large volume dataset, the COVID-19 Genome Sequence dataset [17]. It concluded that non-relational databases outperform SQL databases in aspect of data load time. Moreover, it indicated that non-relational queries were easier to formulate than SQL ones. This has been further supported by another study of a dataset of COVID-19 patients, where the NoSQL MongoDB database showed superior performance over other databases, demonstrating that it is more appropriate for processing large amounts of data [8].

In terms of privacy and security, not only do most non-relational databases not provide encryption mechanisms to protect user-related sensitive data, but also by default the inter-node communication is not encrypted for data in transit [79]. A recent review of advancements for these databases to improve the security reported their use of Kerberos (a computer-network authentication protocol [65]) to authenticate clients and data nodes. It also proposed solutions to deal with remaining shortcomings such as usage of an Identity Provider to authenticate and communicate where the user needs to login using a Single Sign-on method [91]. In addition, researchers have designed a Security-as-a-Service model for NoSQL databases (SEC-NoSQL) which supports execution of query over encrypted data with guaranteed level of system performance [75].

### Data storage costs and cloud implementation

Another important aspect when comparing different types of databases is the costs of running the database; this is particularly significant for large organisations which deal with large volumes of data on a daily basis. Focusing on financial trading data, four different databases were used for comparison in [53]. While MongoDB proved the fastest to read and write end-of-day OHLC (Open, High, Low, Close) data — the SQL solutions were 1.5 × to 3 × slower — in terms of costs MongoDB was definitely the most expensive due to its commercial licensing costs.

To reduce costs, more and more databases run on cloud platforms as they offer low-cost servers and high-bandwidth networks delivering better reliability, durability, scalability and accessibility of data [2, 15]. As mentioned before, as scalability is a particular strength of non-relational databases, their presence on Cloud allows their growth in a matter of just a few clicks [1]. Not only do the main cloud providers support and manage a variety of relational databases (such as the popular Oracle, MySQL, and PostgreSQL), but they have also been developing their own proprietary non-relational databases to address their own needs, e.g., BigTable by Google or DynamoDB by AWS (Amazon Web Services) [25]. Indeed, for example, in 2006, Google needed a solution for its ever-growing collection of semi-structured data that was distributed across multiple data centres worldwide. As the relational model they had been using was unable to accommodate such a large pool of data efficiently enough, they created BigTable, a document-based database. Nowadays, it handles most of their infrastructure [18]. Advancements in non-relational architecture motivated Yahoo to develop criteria to quantitatively evaluate non-relational database systems. Its Cloud Serving Benchmark is the most widely used and well-known benchmarking framework for evaluating NoSQL databases with varying workloads [16, 22, 96].

In [26], the author has surveyed non-relational databases on Cloud and recorded their features in terms of the storage type (Column, Key-value, Document or Graph), the license type (Commercial or Open source) and the programming language used to develop them. He reported that, out of the 15 cloud databases surveyed, MongoDB, Cassandra and HBase were the most used.

The research paper by Fang at el. [29] show how financial markets have evolved in the last decade and have become more complex and interconnected than ever before. One cannot get a comprehensive view of a portfolio with one source of data. In the financial markets the volume of the data grows exponentially: with the growing capabilities of computers, many companies have used a fast-increasing amount of historical data to feed predictive models, forecasts, and trading impacts. Advances in big storage and processing frameworks combined with the cloud capabilities have helped financial services firms to unlock the value of data, improve their volumes and, commissions, and reduce the cost-of-trades [39]. Moreover, a recent survey has shown the value of ‘alternative data’, i.e., data originating from non-financial sources such as social media, GPS, or sensor data, for predicting stock prices and discovering new price movement indicators [40]. Consequently, capital firms need to store and stream, in various formats, enormous amount of data, and effectively link the data together to get an actionable insight. Big data processing frameworks, which offer parallel and distributed algorithms running on clusters of servers such as MapReduce [24], Hadoop [90], Spark [98], have fulfilled their requirements at least in terms of carrying out their batch processing tasks [80]. With the increase in computing power and decrease in data storage costs, collecting and processing large amounts of data has become an increasingly viable and exercised routine in the financial industry. Still, it is important for such organizations to select their database carefully so that it can, not only store and process big data, but also handle their growth in the long term.

As previous studies have shown, no database system provides best performance in all scenarios. On one hand, relational databases deliver accuracy and redundancy by following the ACID properties. On the other hand, non-relational databases support large and distributed datasets with frequently changing schemas providing better performance and flexibility [73], which makes them particularly attractive for industries requiring high-performance analytics capabilities and distributed large data scalability [49]. Currently, efforts are being made to merge the two database systems to offer the best of both worlds [45, 92], where, for example, a hybrid model would provide the flexibility that is prevented by the rigid relational database framework [54]. Most recently, a hybrid database was implemented where simple requests (read, insert) were served by MongoDB, while complex operations, such as joins with filtering the requests, were forwarded to PostgreSQL [43]. These hybrid models integrate SQL and NoSQL databases in one system to eliminate the limitations of individual systems. Even though they have produced promising results, their adoption has hardly started. Indeed, not only do they make maintenance more complex as two different databases must be handled, but also their associated costs are added. Moreover, a hybrid interface must be written to bridge the two databases together. Finally, there is no readily available solution that an organisation can install and run like any other database system.

Considering all the limitations of database systems when dealing with big time-series data and the requirement to use a system that can scale on-demand, in the next section we will be proposing a set of criteria to consider when selecting a database. We will then use a custom benchmarking tool for recording the results of our experiments and rating each database against the criteria to propose the best performing database.

## Hybrid cloud architecture for secure and efficient storage and processing of financial data

The state-of-the-art review shows that even though relational databases have been the standard storage systems over the last four decades, recent advancements in alternative database technologies have put into question the status quo. As the exponential increase in data volume, velocity and variety challenges what relational databases can handle, industries have been turning to NoSQL for data storage and management.

**Fig. 1:**
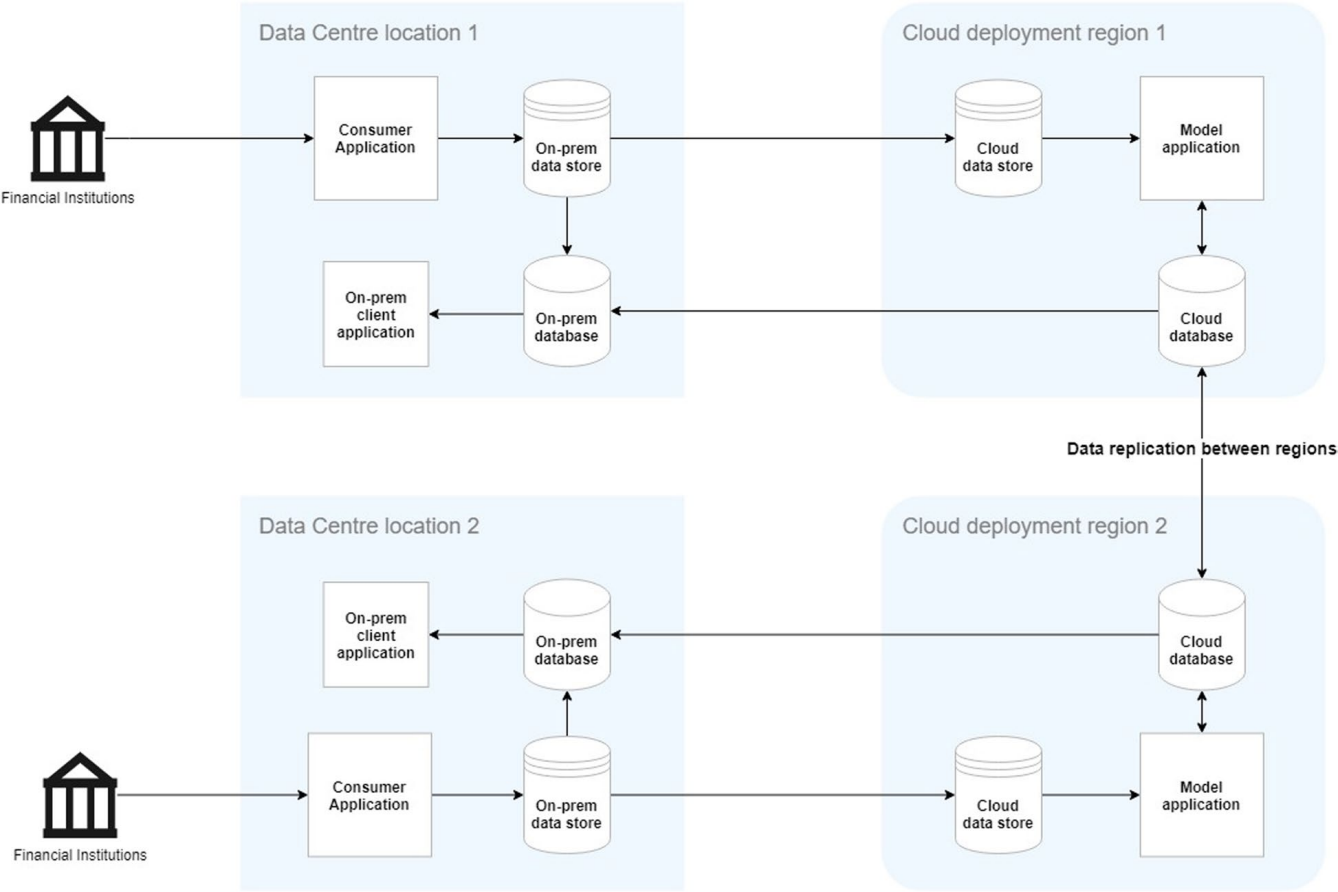
Hybrid cloud deployment architecture

Many large organisations including those from the financial industry have elected a hybrid cloud strategy [29], i.e., a combination of a public cloud with on-premises (on-prem) data centres. The scale, power, and flexibility of the hybrid cloud provides financial companies with significant benefits, particularly the ability to extend existing infrastructure without incurring large capital costs while retaining latency prone applications and sensitive data/code on-premises as appropriate or mandatory by regulations. Moreover, these international organisations take advantage of cloud databases to replicate and distribute data immediately to multiple geographic regions thus offering real-time data access worldwide. Users no longer have to deploy middleware to deliver database requests anywhere in the world, as clearly depicted in the following architectural diagram (Fig. 1). As shown in the figure, the data produced from the financial institutions in the region is consumed by the applications running in the nearest data centre and is stored on the on-prem data store, from where it is transferred to the cloud and processed for model training and inference. The results are then stored on to a cloud database and replicated over the cloud among various geographical regions, making it easy and fast to query for the users, possibly located thousands of miles apart from the original source of the data.

With over 200 solutions available, choosing the right database for a given use-case is particularly difficult. In this article we suggest a set of criteria to ensure adequate storing and querying of OHLC financial data. This selection is guided by the requirement to query multiple records from the database in a high throughput scenario where speed is critical. In addition, such performance should be achieved with minimal data storage and maintenance costs to the organisation. These criteria are listed below.

1. *Database scalability*. Top level databases are distinguished by their ability to grow the capacity of the database on demand. In traditional databases, expansion is achieved by replacing the existing storage or server with a bigger server. As seen in the previous section, even the biggest affordable servers might not be able to meet storage requirements of data volume increasing exponentially [10, 83], which leads to either restriction of rapid data expansion or a single point of failure. On the other hand, databases that support partitioning of data across servers, which is often referred to as horizontal scaling or scaling out, allow increasing storage requirements at minimal cost.
2. *Data model and throughput*. As revealed in the literature review the primary difference between the SQL and NoSQL databases is that the latter promote flexible design by not using relational data models [50]. Such flexibility allows the design of much simpler and cheaper alternatives which can deliver high efficiency and throughput (transactions per second). Moreover, unlike relational databases which rely on table schema, NoSQL’s flexible nature makes it also a lot easier to add new fields and attributes to the data. On the other hand, relational databases are useful when the relationships between different entities need to be explicitly expressed.
3. *Elasticity*, i.e., the degree to which a system can adapt to workload changes by provisioning and deprovisioning resources. The elasticity of a system determines how responsive it is to current demand, affecting directly performance and costs. Cloud managed databases are more elastic compared to the user-managed systems as indicated in our review [1], since these systems can auto-scale both in terms of compute (number of CPUs/cores) and storage. In principle non-relational databases benefit from this by scaling horizontally, this then allow faster retrieval of data as the load is distributed among the new servers.
4. *Maintenance of databases*. A secure and efficient database system must keep up with the latest changes, bug fixes and security patches. With cloud managed databases the maintenance is completely outsourced as the cloud providers automatically update instances to ensure that the underlying hardware, operating system, and database engine are reliable, performant, secure and up to date [15]. Operational costs can be greatly reduced for organisations using cloud managed databases as they are easier to provision, update, and scale along with being more reliable (with almost no downtime) and secure.
5. *Storage efficiency and costs*. Efficient storage permits limiting the amount of space required to store data, which reduces storage costs. Moreover, by accommodating rapid data growth, it can ensure sustained performance regardless of the size of the database [26]. As cloud databases use next generation I/O (Input–output) optimized storage drives, they can provide faster access to the data for only a negligible amount of extra costs.

**Fig. 2:**
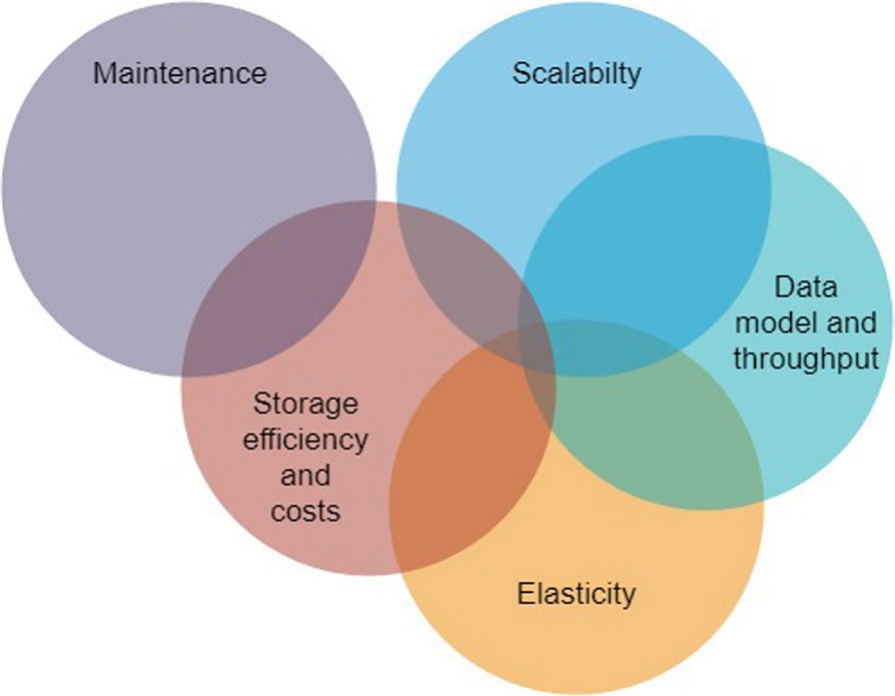
Criteria chosen to evaluate database performance and their overlaps

There are relationships and overlap (see Venn diagram in Fig. 2) between these individual criteria which often sees an improvement in one coming at the expense of another. For instance, scalability, elasticity, and maintenance directly affect the database running costs and so does the database storage efficiency.

**Fig. 3:**
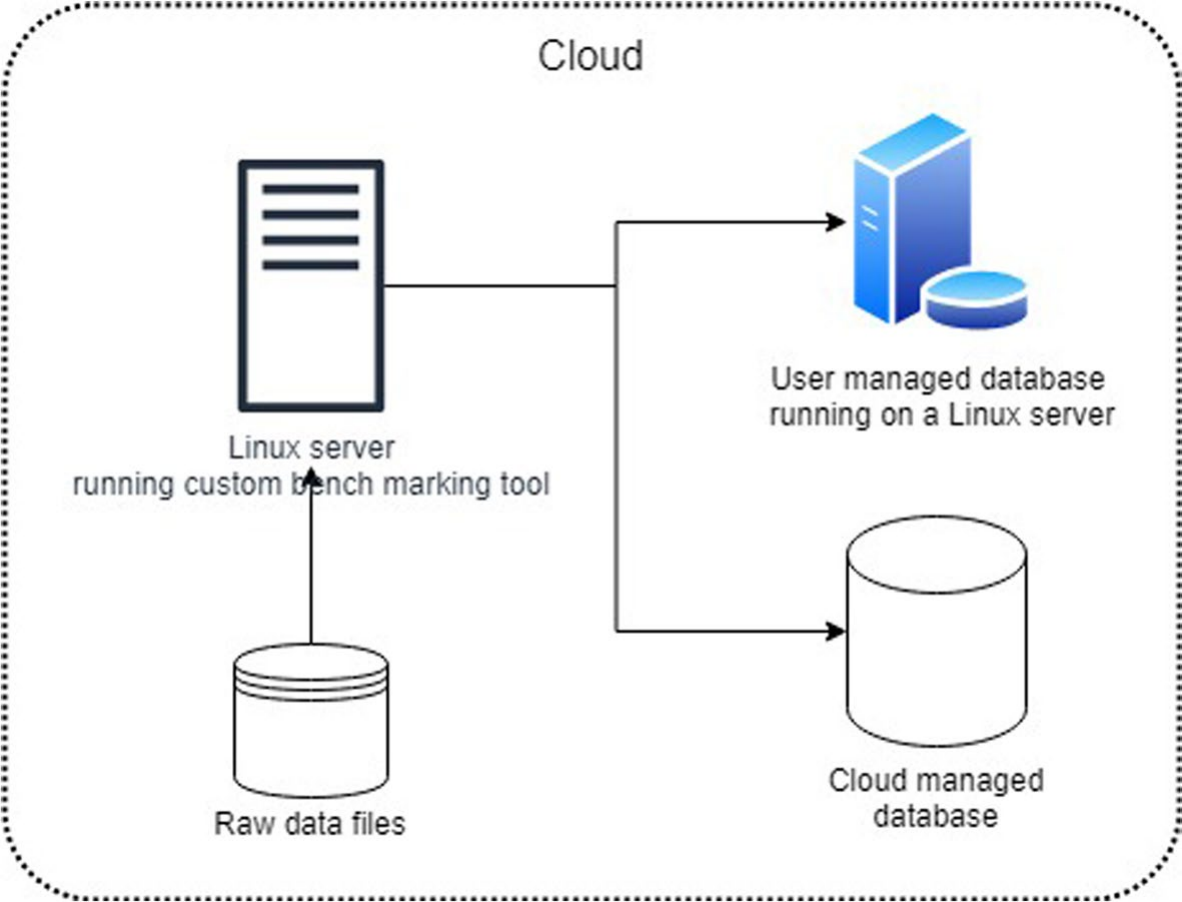
Architecture used to conduct our experiments

In the next section we carry out experiments to evaluate the performance of various database systems in terms of the above-mentioned criteria. Given the structured nature of the OHLC data, the flexibility of the data model is less important in this analysis. Figure 3 shows a block diagram of how our custom benchmarking tool [14] operates to carry out the experiments with databases running on the cloud. The dataset is first downloaded and stored on to a cloud data store from where it can be easily accessed by the Linux server running our benchmarking tool. This data is then loaded on to the databases running on either a user managed server or as a service managed by the cloud provider. Our conclusion will be based on how well these databases perform when run against various data workloads. Finally, we will propose the best performing database as an optimal choice to store the ever-growing OHLC financial data.

## Experiments and results

After specifying the cloud environment and the databases selected for the experiments, this section presents the dataset and the evaluation framework that have been used. Then, it reports and analyses the results for all the considered benchmarking workloads. Finally, a discussion leads to the identification of the type of database that is the most suitable to store and query OHLC financial data.

### Cloud environment and databases

The experiments we present in this paper have all been executed on the cloud as it offers flexibility to run database workloads both traditionally (on a virtual server) or as a service (managed by the cloud provider). More specifically, the cloud delivers large storage, high throughput, and low latency capabilities. In addition, it offers scalability when the load increases. As per the recent Gartner (world’s leading research and advisory company) report [11], the top three leaders in cloud computing in order of their popularity are Amazon, Microsoft, and Google, where the leader, Amazon Web Services (AWS), holds about 40% of the market share providing some of the best services to choose from. In particular, it offers a wide range of database services including relational, non-relational, hybrid and time series databases. AWS is therefore the choice of cloud provider for the experiments performed in this study.

AWS offer their users a choice between running a self-managed instance of database on a virtual compute server (EC2 – Elastic Compute Cloud) and their Database as a Service, Amazon Relational Database Systems (RDS) which supports six database engines –MySQL, PostgreSQL, MariaDB, Oracle, Microsoft SQL Server and Amazon’s own cloud-native RDBMS, Aurora. Both Amazon EC2 and Amazon RDS offer different advantages. Amazon RDS is easier to set up, manage, and maintain than running a database on Amazon EC2. This lets the user to focus on other important tasks, rather than the day-to-day administration of the database. Although running databases on an Amazon EC2 is uncommon because of the lengthy process of setting up and maintaining the infrastructure, it provides a secure, resizable compute capacity in the cloud giving the user more control, and flexibility over the resources. Both Amazon EC2 and Amazon RDS have an associated storage volume called Elastic Block Store (EBS). EBS offers a high-performance block-storage which is easy-to-use, highly available, durable, and scalable. A recent study by International Data Corporation (IDC), a premier global provider of market intelligence, advisory services, and events for the technology markets [44] found that the customers using RDS had 39 percent lower database operation costs and 264 percent return on investment over three years. When it comes to the non-relational AWS managed databases, diverse data models are supported including key-value (e.g., DynamoDB), document (DocumentDB), in-memory (ElastiCache), graph (Neptune) and time series (Timestream).

For this experiment, the most popular and widely supported databases in each of the categories were selected. Note that commercial databases, such as Oracle and Microsoft SQL Server, were not considered due to licensing constraints. For the relational databases, the opensource MySQL and PostgreSQL were chosen. Data workloads were first run with databases running on EC2s and the results were recorded into a CSV (Comma separated values) file. RDS MySQL and RDS PostgreSQL databases were then used for running the workloads followed by the NewSQL/Hybrid database by AWS – Aurora MySQL and Aurora PostgreSQL. All these databases use the same version of MySQL (version 5.7) and PostgreSQL (version 13.3) respectively. The same pattern was followed for non-relational databases, by running the data workloads first with MongoDB running on an EC2, followed by DocumentDB (AWS managed Document Database that supports MongoDB workloads) [97], and finally DynamoDB (serverless AWS managed NoSQL database) [19].

For all our experiments, the server-based databases were run using the r5.2xlarge (db.r5.2xlarge for AWS managed) instance type which includes 8 vCPUs and a 64 GBytes RAM (Random Access Memory) running on an Amazon Linux 2 AMI (Amazon Machine Image). Although these are modestly sized instances, especially when it comes to the memory requirements, it is sufficient to conduct this study’s experiments. Each database was allocated its minimum required SSD (Solid State Drive) storage, i.e., 8GBytes for user-managed databases running on EC2s, 10GBytes for Aurora and DocumentDB, and 20GBytes for RDS instances. On the other hand, as DynamoDB is a serverless database, it has the ability to scale up or down its required resources based on the demand. Therefore, there is no fixed compute or storage for DynamoDB.

In addition to the above resources a virtual compute server or an EC2 is used to run the benchmarking tools. The EC2 instance type is a t3.2xlarge instance with 100GBytes SSD volume to store the datasets. The scripts are run on an Amazon Linux 2 AMI with Java SDK8 (Software Development Kit) and Python3.6 installed on top.

### Data

There are a number of online sources (including Google Finance, Yahoo Finance) that provide OHLC data. The data set used in this study has been sourced from Kaggle, an online community of data scientists which provides financial data at no cost. Kaggle is a popular data provider that has been used in many of the research projects [4, 42, 84]. Specifically, this study uses the “Huge Stock Market Dataset” [62], which provides millions of historical data points across a broad range of instruments. This study will be using the historical daily price and volume data for securities on the NYSE (New York Stock Exchange) and NASDAQ (National Association of Securities Dealers Automated Quotations). The dataset contains the OHLCV (OHLC along with the total traded volume) values for up to 7,195 stocks and 1,344 ETFs (Exchange-Traded Funds) for each day between 1968 and 2018 (according to their availability on the market), i.e., a total of 12,648 trading days. For each security there is a single file named after its ticker symbol which contains one record per day starting from the day the security was first listed on the exchange until either its last day on the exchange or the end of 2018. Each record contains six values: the date, open price, high price, low price, close price, and the volume. Table 1 shows a sample of records for ‘Apple’ in the year 1984 when it was first listed on NASDAQ. Prices are represented by numbers with five decimal places. The data is in its raw format without any transformations besides adjustments to the prices for dividends and stock splits to provide a more accurate measure of the real value of the stock [4].

**Table 1:** Sample input data for apple (AAPL)

Date	Open	High	Low	Close	Volume
1984–09-07	0.42388	0.42902	0.41874	0.42388	23,220,030
1984–09-10	0.42388	0.42516	0.41366	0.42134	18,022,532
1984–09-11	0.42516	0.43668	0.42516	0.42902	42,498,199
1984–09-12	0.42902	0.43157	0.41618	0.41618	37,125,801

### Evaluation framework and scope

Performance comparison was achieved by loading the “Huge Stock Market Dataset” to the selected databases, performing queries, and running various workloads. This was informed by the workloads defined in the Yahoo! Cloud Serving Benchmark (YCSB) [22], the most popular benchmarking framework for NoSQL databases. The YCSB tool could not be used as it does not have support for traditional relational databases. Therefore, to ensure benchmarking consistency, we designed our own custom scripts [14] to interact with all databases. Our custom benchmarking tool was developed using well-documented libraries in Python and, similar to YCSB, it comprises of two components: a data generator where the data is loaded on to the database (load operation), and a workload generator which runs predefined workloads (run operation) based on parameters such as percentage of inserts, reads, updates or deletes, as well as the number of operations and the number of records. To ensure a fair comparison all databases were evaluated by running the same set of queries with the predefined workloads, also maintaining the query execution order.

Due to the application requirement of dealing with real time data, an appropriate database needs to be able to load the selected dataset with at least 250 k inserts/sec. For such a high throughput it should allow batch inserts and reads, and usage of multiple threads or concurrent connections. This is particularly important to take advantage of the parallel processing power of the system on which the analysis is running. Moreover, as OHLC data is time series data, their analysis requires performing rapid operations such as aggregations, filtering, and joins on the date and time fields.

This experiment has been conducted using a small dataset involving two exchanges. In real-world applications, analysis is run on much larger data sets which is multiple-folds the size of the experiment. Thus, an appropriate system must be able to scale out to support huge datasets (multiple terabytes or even petabytes) and very high request rates. Fortunately, most of the cloud native databases (including RDS, Aurora, DocumentDB, DynamoDB) were architected to scale-out by distributing load across multiple servers [5].

Although in a production workload, high availability of the data and disaster recovery provisions are essential, these aspects are not considered in this study since they are delivered by cloud databases that store database snapshots and support multiple replicas over multiple regions [59]. Instead, the focus of this study is to measure latency and throughput in terms of reads, writes and updates over time as the data grows, database scalability and elasticity (i.e., the ability to adapt to changing workload by provisioning and deprovisioning resources), storage space usage, and associated costs [61].

### Experimental results

This section presents the results obtained after running the predefined workloads with each of the selected database systems. For running the experiments, the dataset was divided into two equal parts — ‘load’ and ‘insert’. Whereas the ‘load’ data were solely used to load the data in the database before running the workloads, the ‘insert’ data were exploited to perform insert operations when running the workloads. For these experiments a total of five workloads were defined with varying proportions of ‘insert’, ‘read’ and ‘update’ operations as shown in Table 2. Note that the values of the first 4 workloads were chosen to emulate the default workloads defined in YCSB [22]. Moreover, an additional balanced load was included.

**Table 2:** Benchmarking workloads

Workload	Insert (%)	Read (%)	Update (%)
a	0	95	5
b	0	50	50
c	50	50	0
d	50	0	50
e	33.3	33.3	33.3

**Fig. 4:**
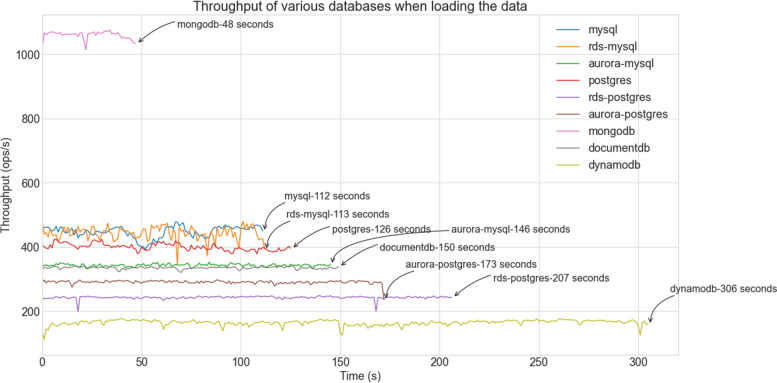
Performance of databases with ‘load’ operation

The initial load operation was performed with 100,000 data records on each of the database systems. During the process, throughput (operations/second) and maximum, minimum, and average latencies over fixed time intervals were recorded. Analysis of the associated graph, Fig. 4, shows that MongoDB outperforms all other systems in terms of required loading time. It was able to load the whole subset of data in 48 s, whereas the runner-up MySQL took more than twice the time, i.e., 112 s. We note similar performance of MySQL running on either user managed EC2 or AWS managed RDS instance, whilst Aurora MySQL requires 29% more time. This may be explained by the fact that Aurora offers high availability by storing multiple copies of the data in different availability zones. While the PostgreSQL database on EC2 only requires 12% more time than its MySQL equivalent, its RDS and Aurora versions deliver much poorer performance. Eventually, the AWS proprietary DynamoDB database proves to be the slowest system, taking around five times longer than MongoDB. This behaviour will easily be explained by the following analysis of the size of the created tables.

Table 3 presents the size of the tables created for each of these database systems. As expected, there is good correlation between table size and data loading time: MongoDB’s is the most compact, while DynamoDB requires more than 24 times more space. Indeed, DynamoDB stores most of the data in form of a character string.

**Table 3:** Database table size

Database	Size (MBytes)
mongodb	4.93
mysql	6.52
rds-mysql	6.52
aurora-mysql	6.52
postgres	8.35
rds-postgres	8.35
aurora-postgres	8.35
documentdb	25.67
dynamodb	120.00

**Fig. 5:**
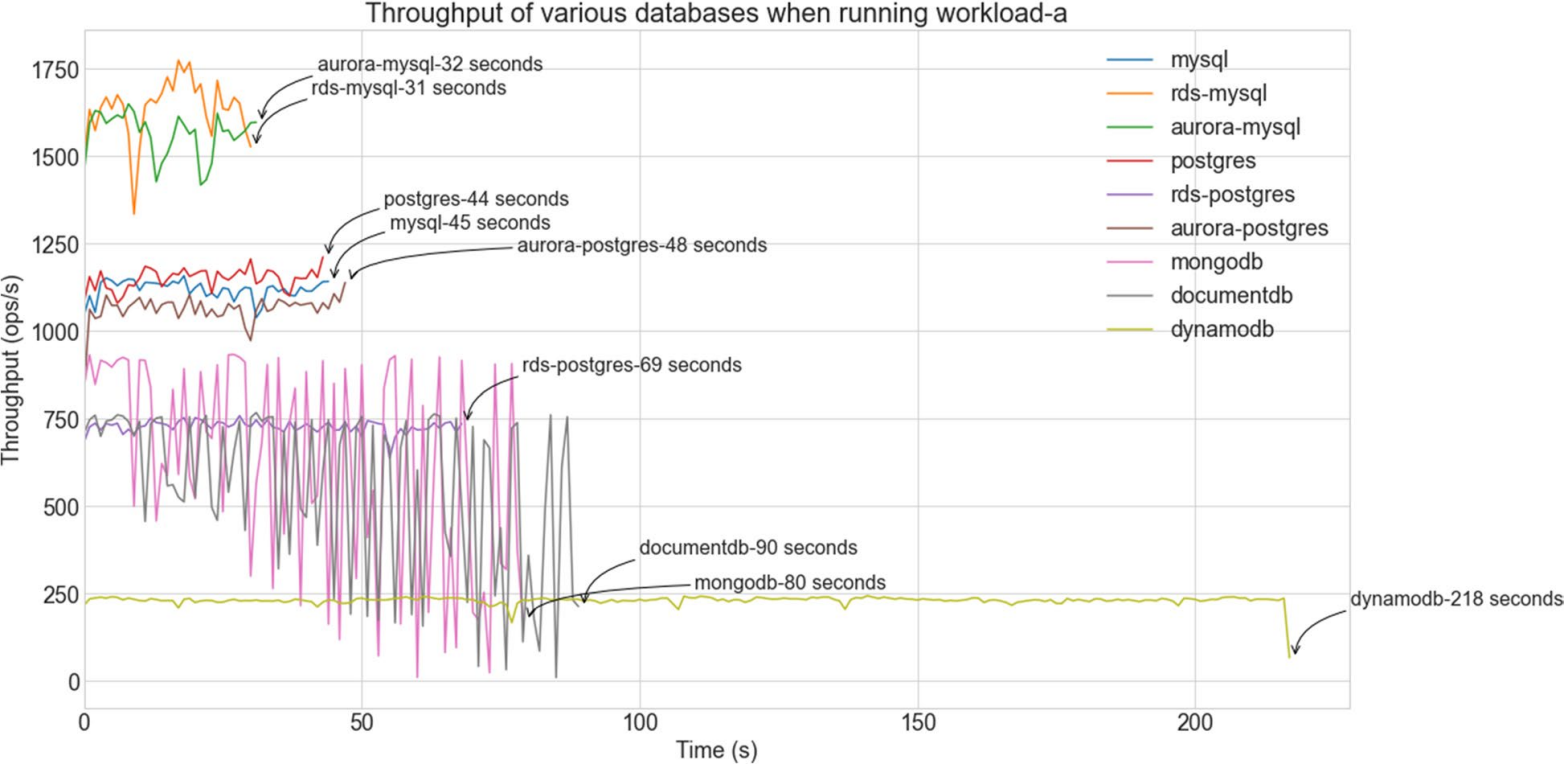
Performance of databases with Workload-a (Read-intensive tasks)

**Fig. 6:**
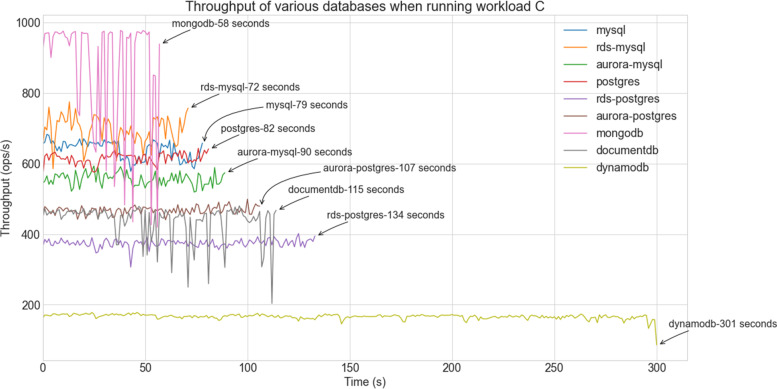
Performance of databases with Workload-c (Read & Write Intensive tasks)

Table 4 shows the average values of maximum, minimum and average latencies (and standard deviations) in milliseconds for all databases in a two-second interval. The average and the minimum latency values are in line with the graph displayed in Fig. 4: while MongoDB requires less than 1 ms to perform one ‘insert’, DynamoDB takes about 6 ms. The maximum latency values (also visible on Figs. 5 and 6) vary widely from database to database. Such variations may be associated to various phenomena, including the locking strategy employed by MongoDB databases, the latency observed while waiting for data to be replicated to redundant instances for high availability of RDS databases, and the presence of abnormal traffic load. Despite high maximum latency values, standard deviations are quite low indicating that those maxima should be seen as outliers.

**Table 4:** Latency statistics

Database	Max Latency (ms)	Min Latency (ms)	Avg Latency (ms)	Std Deviation (ms)
mongodb	2.83	0.82	0.85	0.28
mysql	9.36	1.62	2.13	0.74
rds-mysql	9.33	1.10	2.15	0.71
aurora-mysql	7.48	2.14	2.80	0.63
postgres	8.32	2.13	2.40	0.86
rds-postgres	9.14	3.53	3.81	0.95
aurora-postgres	7.74	3.04	3.32	0.31
documentdb	7.44	2.56	2.88	0.32
dynamodb	17.63	5.36	6.01	2.74

Following the presentation of load performance, which has shown the dominance of MongoDB, the focus moves on the evaluation of the various systems under the predefined workloads. One should note that for us, the most important ones are workload-a (read-intensive) and workload-c (read & write intensive), as in our use case the dataset is rarely updated. Figures 5 and 6 show the performance of the databases in terms of their throughput for workload-a and workload-c respectively.

For the read intensive workload-a, the AWS managed MySQL databases perform best completing the 100,000 operations in about half a minute. This may be explained by the throughput optimized storage that these instances use. The SQL instances running on EC2s require up to 45% more time. While AWS managed PostgreSQL instances, i.e., aurora- and rds-postgres, are less efficient, the NoSQL databases are those which perform worse. In particular, DynamoDB needs a total of 218 s to finish the task. Such slow processing is likely to be the outcome of the data partitioning used for storing a database on multiple nodes.

However, experiments with workload-c reveal a similar picture with the exception of MongoDB which performs best. Also, as workload-c consists of a set of randomly arranged read and write operations, most systems deliver a throughput with large variations. In the case of MongoDB, the ‘reads’ have much higher latency than the ‘inserts’. Note that similar results were obtained for the remaining three workloads that include a higher proportion of update operations. Their associated graphs can be seen in the appendix of this manuscript.

In terms of cost comparison, Table 5 provides estimates of what it would cost to load the data and run workload-a on each database assuming the database is shut down after the operations finish. As seen in the table, these databases have different minimum storage requirements: while RDS databases require a minimum of 20GBytes to be pre-provisioned on creation, Aurora and DocumentDB instances need a minimum of 10GBytes, and for the databases running on an EC2, the root volume size should not be less than 8GBytes. Along with a minimum storage these instances also have a minimum billing time, it is 60 s for the EC2s and 600 s for RDS, Aurora and DocumentDB. The costs are calculated using the following formula

**Table 5:** Running costs (as on 1^st^ December 2021)

Database	Database Type	Compute costs (USD-per-hour)	Storage (GBytes)	Storage costs (USD per GByte-month)	Billing Time (secs)	Total Costs (USD)
mongodb	Customer managed	0.592	8	0.116	128	0.021
mysql	Customer managed	0.592	8	0.116	157	0.026
rds-mysql	AWS managed	1.12	20	0.133	600	0.187
aurora-mysql	AWS managed	1.36	10	0.10	600	0.227
postgres	Customer managed	0.592	8	0.116	170	0.028
rds-postgres	AWS managed	1.176	20	0.133	600	0.197
aurora-postgres	AWS managed	1.36	10	0.10	600	0.227
documentdb	AWS managed	1.30	10	0.116	600	0.217
dynamodb	AWS managed serverless	0.78	0.12	0.03	524	0.113

$$\left(\frac{c}{3600}+\frac{s*g}{30*24*3600}\right)* t$$

where,

c is the compute cost in USD-per-hour (United States Dollar-per-hour) 

s is the allocated or the used storage size in GBytes 

g is the storage costs in USD per GByte-month (here it is assumed that a month has 30 days) 

t is maximum of either the minimum billing time or the time it took to finish the execution of the load operation and workload-a, in seconds.

Table 5 reveals that, as AWS is solely responsible for the maintenance, including upgrades without downtime and operational tasks like automated backups, failover, high availability and durability of all RDS instances including the MySQL, and PostgreSQL and the DocumentDB, their costs are up to 10 times those of the user managed instances. Still, in the long-term AWS managed databases would be a good choice if outsourcing the maintenance work was seen beneficial by reducing operational costs even at additional running costs. Moreover, since Aurora instances offer additional functionality such as storage auto-scaling and high availability, their costs are even higher. Finally, DynamoDB with provisioned capacity of 1000 read units and 1000 write units would cost about $0.134, which is cheaper than any other AWS managed databases used in our experiments.

Finally, we review the possible solutions using the set of *criteria* previously defined. Our experiments show that, in terms of *Database scalability*, a customer-managed instance of a database running on an EC2 is not self-capable of scaling up or down based on the demand. An organisation requiring such capabilities would have to opt for an AWS managed database. Regarding *Data throughput*, with the exception of read-intensive tasks, MongoDB outperforms all the other database systems we investigated. Alternatively, when considering read-intensive workloads, SQL databases, in particular MySQL, seem to be the solution of choice as they consistently deliver good throughput in all evaluated scenarios. On the topic of *Elasticity*, self-managed database instances require a manual intervention to add or remove new nodes based on the demand, whereas an AWS managed instance can deliver such services without such intervention. One of the useful features of using Cloud technologies is that it offers autoscaling of resources which can be triggered based on the utilisation metrics of resources. This needs to be manually configured for the customer-managed instances whereas it is automatically provided with AWS managed databases. With respect to *Maintenance*, operational tasks, such as updates, security patches, and encryption, are automatically performed by AWS for all the managed databases, whereas these tasks need to be carried out manually for customer managed instances. In addition to requiring in-house expertise and resources, the latter are often associated with delays and downtimes. Lastly, in terms of *Storage efficiency*, MongoDB is the most economical followed by the SQL databases. However, it is important to specify that in addition to the data volume, the maintenance type and throughput directly affect the database running costs. Although DynamoDB, the AWS managed serverless database, performed worst in our experiments, AWS claims that some substantial performance improvements (up to 10 × faster) might be observed by introducing a caching layer (DAX – DynamoDB Accelerator [85]) in front of the DynamoDB table. We were not able to test this, but one should keep in mind that would come with additional costs and would only impact the ‘read’ operations.

Regarding our specific case study which involves storing and querying an essentially linearly growing (as historical data is never discarded and variations of the number of stocks are limited) volume of OHLC financial data, our experiments suggest that MongoDB is the best choice. Firstly, in all tested configurations with the exception of Workload-a, it delivers best performance. Secondly, it is also the cheapest, which is particularly important as data records permanently increase in number. Indeed, MongoDB has ability to efficiently compress data, which directly reduces the costs of running and maintaining them. Thirdly, being a NoSQL database MongoDB can be scaled horizontally by adding more servers to the network, which makes it more elastic compared to the SQL databases. Naturally, this would come at the expense of spending extra time and resources on maintenance. DocumentDB would be an interesting choice for an organisation wishing to outsource these operational tasks. Although MySQL offers better ‘read’ performance, it still lags behind MongoDB for all other types of workloads. Moreover, low latency and high throughput are not the only requirements here. As specified earlier: scalability, elasticity, storage, and costs all contribute to our decision. Since MySQL is a relational database, it cannot scale horizontally. Moreover, it occupies more storage space and is costlier than MongoDB. Therefore, a relational database like MySQL would not be an optimal choice especially when we are talking about Peta-byte range of data [74].

## Conclusion

As more and more financial organisations run their production load in hybrid cloud environments and many alternatives to the standard relational databases are available, the selection of the most appropriate database has become more challenging. To address this, this study evaluated performance of popular relational and non-relational databases for storing and querying financial Open High Low Close data. This was performed by conducting experiments whose outcomes were analysed according to a set of application-relevant criteria, i.e., scaling power, throughput and latency, elasticity, maintenance, storage space used and their associated costs.

Those experiments revealed that the non-relational databases are fully capable of replacing the relational databases traditionally used for storing OHLC data. In particular, MongoDB offers best performance in terms of query execution time for most of the considered workloads while consuming only a fraction of storage space used by relational databases. Moreover, unlike relational databases, it is also very responsive to both the ever-growing data flow and highly variable workloads. Finally, we suggest that, in the context of a hybrid cloud environment, organisations would benefit from the additional capabilities offered by cloud-native databases since they offer valuable services such as automated maintenance, automatic backups and replication including cross-regional replication and creating read-replicas of the database, which delivers higher availability. In further work, it would be interesting to evaluate the responsive power of these cloud-native databases in terms of scalability, when data outgrows the storage capacity, and also elasticity by varying the load on the database.

## Figures and Tables

**Fig. 7:**
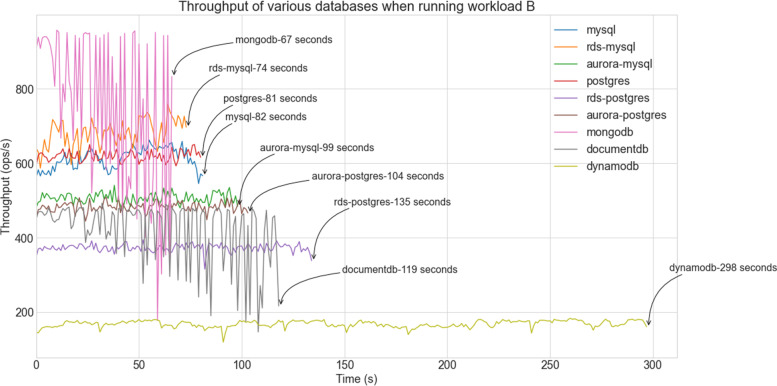
Performance of databases with Workload-b (equal number of reads and updates)

**Fig. 8:**
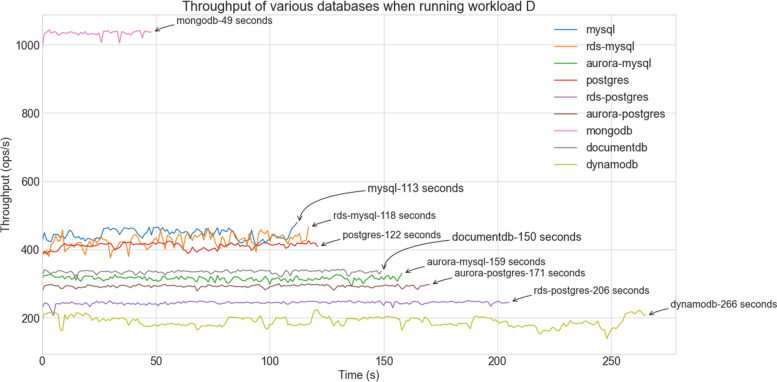
Performance of databases with Workload-d (equal number of inserts and updates)

**Fig. 9:**
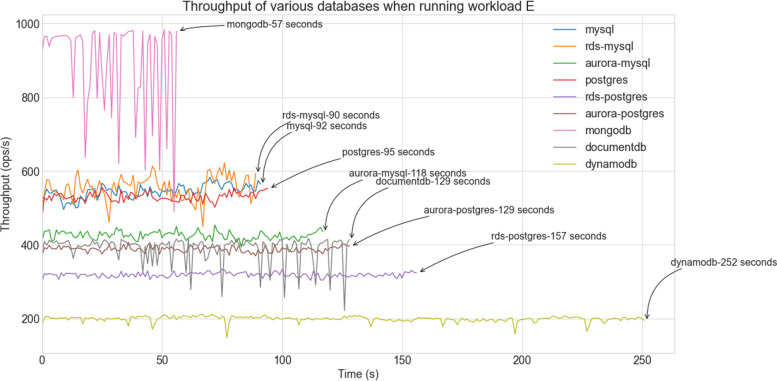
Performance of databases with Workload-e (equal number of read, inserts and updates)

## Data Availability

The dataset supporting the conclusions of this article is available in the Kaggle datasets repository, [https://www.kaggle.com/borismarjanovic/price-volume-data-for-all-us-stocks-etfs].

## Appendix

Fig. 7 

Fig. 8

Fig. 9

## Abbreviations

AAPL: Apple
ACID: Atomic, Consistent, Isolated, Durable
AMI: Amazon Machine Image
AWS: Amazon Web Services
BASE: Basic Availability, Soft state, Eventual consistency
CAP: Consistency, Availability, and Partitioning
CPU: Central Processing Unit
CSV: Comma Separated Values
DAX: DynamoDB Accelerator
EBS: Elastic Block Store
EC2: Elastic Compute Cloud
ETF: Exchange Traded Fund
GBytes: Gigabytes
IDC: International Data Corporation
IoT: Internet of Things
I/O: Input–Output
KTP: Knowledge Transfer Partnership
Mbytes: Megabytes
NASDAQ: National Association of Securities Dealers Automated Quotations
NoSQL: Not Only Structured Query Language
NYSE: New York Stock Exchange
OHLC: Open, High, Low, Close
OHLCV: Open, High, Low, Close, Volume
RAM: Random Access Memory
RDBMS: Relational Database Management System
RDS: Relational Database Systems
SDK: Software Development Kit
SQL: Structure Query Language
SSD: Solid State Drive
USD: United States Dollar
YCSB: Yahoo! Cloud Serving Benchmark

## Footnotes

1 The open price is not always equal to the close price of the previous trading day. Sometimes events such as company earnings reports or stock splits can happen in after trading hours and can alter a stock’s price overnight.

2 Note that as non-relational databases cannot use SQL as their query language, they are often referred to as NoSQL; in this manuscript, both terms are used interchangeably.
